# Applications of Latissimus Dorsi Grafts in Reverse Shoulder Arthroplasty

**DOI:** 10.7759/cureus.48469

**Published:** 2023-11-07

**Authors:** Sushrut Bose, Ratnakar Ambade, Yashvi Bhartiya, Vivek R Velagala

**Affiliations:** 1 Orthopaedics, Jawaharlal Nehru Medical College, Datta Meghe Institute of Higher Education and Research, Wardha, IND; 2 Medicine, Jawaharlal Nehru Medical College, Datta Meghe Institute of Higher Education and Research, Wardha, IND

**Keywords:** rheumatoid arthritis, shoulder joint, rotator cuff tears, latissimus dorsi flap, reverse total shoulder arthroplasty

## Abstract

The shoulder joint is a multiaxial joint in the upper body known for its high degree of motion. It is also infamously known for recurrent dislocations compared to other joints. These dislocations are mainly fixed by closed reduction methods like the Hippocrates technique, Stimpson's gravity technique, and the most commonly used modified Kocher's technique. The modified Kocher's technique uses traction followed by external rotation, adduction, and internal rotation. Rotator cuff tears are associated with shoulder joint dislocations. Rotator cuff tears slowly heal and persist for 10-20 years, irrespective of their etiology. When left untreated, fibrosis can set in the joint. After fibrosis, it is repaired with a reverse shoulder arthroplasty. Reverse shoulder arthroplasty allows a greater degree of movement compared to the conventional arthroplasty. In reverse shoulder arthroplasty, the latissimus dorsi tendon is removed from its original insertion and attached to the humerus around the insertion of the deltoid muscle. This change increases the torque and external rotation of the joint and provides better results than the surgeries where the tendon transfer is not done. This article compiles the various etiologies of shoulder dislocation and its treatment, shoulder arthroplasty. It discusses the indications and contraindications of total and reverse total arthroplasty. This article aims to compare conventional shoulder arthroplasty and reverse shoulder arthroplasty. It highlights the advantages of using latissimus dorsi grafts in reverse shoulder arthroplasty in shoulder joint dislocations.

## Introduction and background

The shoulder joint is the most mobile joint in the human body. As a result, it is one of the most unstable joints and, thus, most prone to dislocations due to its high degree of instability [1]. The shoulder joint is a synovial joint of ball and socket variety. It comprises of the bony, muscular, ligament, capsule, and stabilizer components [2]. The bony components comprise the glenoid and humerus. The glenoid cavity is a part of the scapula. It is a dish-like structure and is almost flat [3]. Labrum is a fibrocartilagenous structure that attaches to the glenoid’s rim and gives it a socket-like shape. The second bony component is the humerus. The humerus head is a ball-like structure. The humerus head is in 15-20 degrees retroversion, i.e., it is rotated posteriorly, while the femoral head is in 15 degrees anteversion, rotated anteriorly. At a time, only one-fourth of the humeral head articulates with the glenoid. This bony structure is constitutionally very fragile [4].

The muscular components can be subclassified into intrinsic and extrinsic parts. The intrinsic muscles are those that pass across the shoulder joint. They are the rotator cuff muscles, the deltoid, pectoralis major, teres major, latissimus dorsi, and biceps brachii. The rotator cuff muscles constitute the supraspinatus, infraspinatus, and teres major, which attach on the greater tuberosity in superior to inferior order as well as the subscapularis muscle, which attaches on the lesser tuberosity. The subscapularis is also called the missed or forgotten tendon. Out of these, the supraspinatus is the most commonly injured tendon [5]. The interval between the anterior edge of the supraspinatus and the superior edge of the subscapularis is referred to as the rotator interval. The coracohumeral ligament passes through this interval. The extrinsic group of muscles are involved in moving the scapula and do not cross the shoulder joint. They are the levator scapulae, rhomboids, trapezius, and serratus anterior [6]. It receives its blood supply from the anterior circumflex humeral vessels, the posterior circumflex humeral vessels, and the suprascapular and subscapular vessels. The nerve supply comes from the axillary, musculocutaneous, and suprascapular nerves [7].

It is a significant challenge to treat extensive, non-reparable rotator cuff tears, particularly in juvenile and physically active individuals. By performing the tendon transfer, the biomechanics can be readjusted for the shoulder joint. The change involves balancing the force couple, potentially delaying the onset of osteoarthritis. Despite several transfer methods being available, the latissimus dorsi is desirable for addressing deficiencies in the anterosuperior region and the lower trapezius for deficiencies in the posterosuperior region. Initially, the conventional approach for addressing the posterior-superior tears of the rotator cuff involved the transfer of latissimus dorsi and sometimes teres major. Nevertheless, the lower trapezius transfer has surfaced as a substitute that aligns better with the fundamental aspects of reconstructing the transfer of tendons. Various grafting options have been investigated for addressing anterior-superior rotator cuff tears, including the transfer of the pectoralis major and the use of the latissimus dorsi transfer, occasionally in conjunction with the teres major. Recent studies highlight the preference for latissimus dorsi transfer over pectoralis transfer for reconstruction of subscapularis [8]. This article proposes the need for further research in managing rotator cuff tears, comparing the costs and benefits of reverse shoulder arthroplasty with and without latissimus dorsi tendon transfer.

## Review

Methodology

Eligibility Criteria

The study included all the review articles and original studies that included conventional shoulder arthroplasty and reverse shoulder arthroplasty (RSA) without and with latissimus dorsi grafts. These articles discussed basic shoulder and rotator cuff anatomy, the injuries it can sustain, and the ways to treat it. Articles were excluded if the data in them was not relevant to the topic.

Literature Search Strategy

The research study was conducted by all the authors. The study analyzed research articles from 1956 to 2023. The articles included keywords like "shoulder joint," "rotator cuff tear," "shoulder dislocation," "shoulder arthroplasty," "reverse shoulder arthroplasty," and "latissimus dorsi."

Data Extraction

**Figure 1:**
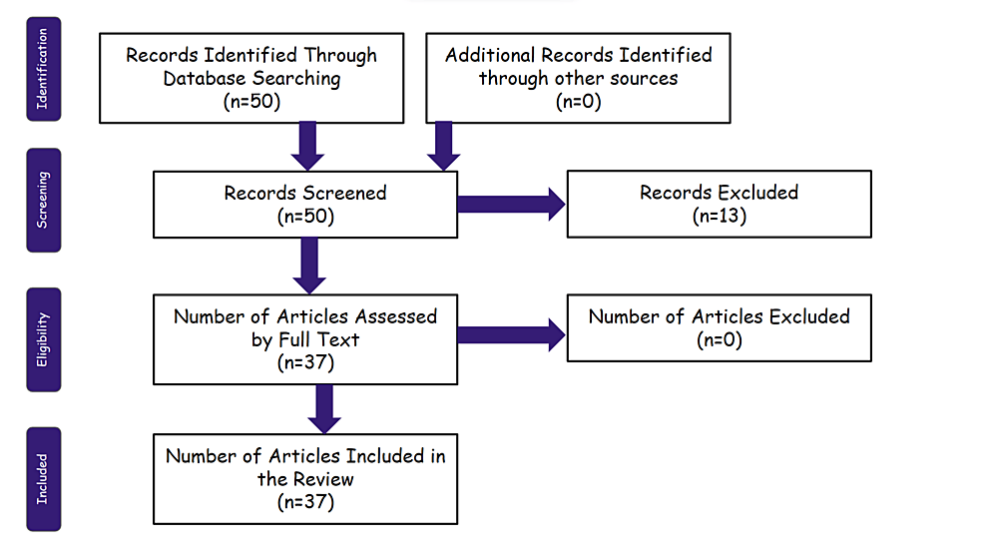
PRISMA flow diagram for screening and selecting articles for the use of latissimus dorsi grafts in reverse shoulder arthroplasty PRISMA: Preferred Reporting Items for Systematic Reviews and Meta-analyses.

The abstracts of all articles were read by the authors independently. The articles meeting the eligibility criteria were studied and assessed for their full texts. The search process is demonstrated in Figure 1.

Shoulder injuries requiring RSA

**Figure 2:**
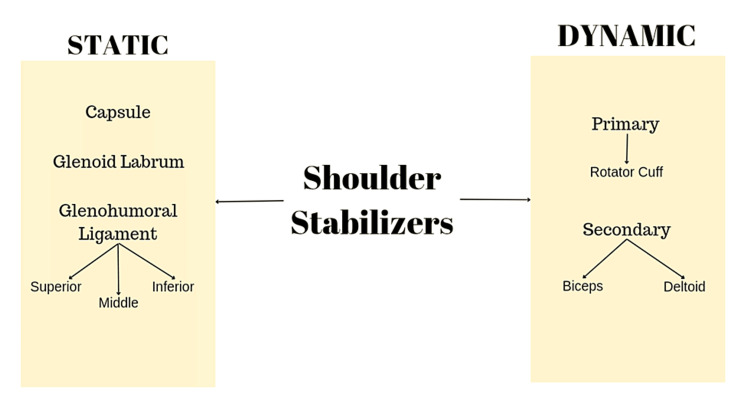
Stabilizers of shoulder joint Image credits: Authors of this study.

Anterior shoulder dislocation is the most common type of shoulder dislocation. A history of trauma will be present whenever a patient comes with this condition. The shoulder joint has two types of stabilizers: static and dynamic. Static type includes capsule and ligaments, whereas dynamic type includes rotator cuff muscles. The shoulder stabilizers are enumerated in Figure 2.

The capsule is weakest inferiorly, yet the most common dislocation is anterior. Anterior type of shoulder dislocation can be subcoracoid, preglenoid, or subclavicular [9,10]. It mostly occurs due to abduction with external rotation. The patient presents with an arm in external rotation and abduction along with loss of contour of the deltoid, which is caused by the absence of an underlying humeral head in the orthopedic position [11]. The anterior axillary fold is seen at a lower level, and the patient cannot touch the tip of the opposite shoulder with the injured hand. The flattening of the deltoid is seen by performing the Hamilton ruler test. It is also seen by X-ray in the A-P view or the Grashey's view [12]. The dislocations will be mostly associated with rotator cuff tears. If not dealt with, the rotator cuff tears will progress and cause fibrosis, leading to cuff tear arthropathy, mostly due to severe glenohumeral arthritis, which will then be treated by a RSA. These patients can also present with pseudoparalysis, meaning they cannot actively elevate their arms [13,14]. There can also be cases where there is malunion or non-union of the bones, for example, the tuberosity following the trauma or a previously performed arthroplasty. It is also done in cases where the previously done arthroplasty has failed, making RSA the last resort [15]. It is also done when the proximal part of the humerus gets fractured in older adults, especially when it involves the head part. This is because these patients will have poor bone quality, reducing the potential for healing [16]. Another cause might be rheumatoid arthritis. But in these cases, the surgery can only be performed if the glenoid bone stock is sufficient [17,18]. A prerequisite for performing RSA in any patient will be a working deltoid muscle, meaning the axillary nerve should be intact. The latissimus dorsi connects the pelvic girdle and the vertebral column to the proximal parts of the upper limb [19]. The origin of this muscle is quite extensive, but it has a narrow insertion. This muscle progresses with its development in the extensor compartment of the limb. Soon after, it migrates to its wide attachment on the truncal area while being supplied by the thoracodorsal nerve. The latissimus dorsi is tested in the posterior part of the fold of the axilla, where the muscle is felt during its contraction by asking the patient to cough [20,21].

Arthroplasty and RSA

Arthroplasty is one of the most common surgical methods to treat shoulder joint injuries. The difference between arthroscopy and arthroplasty is that a long, thin tube is used in arthroscopy to repair the injured parts of the joint, while the complete parts of a joint are repaired in arthroplasty. Arthroplasty may also be done in degenerative disorders like osteoarthritis in advanced stages. Depending on the doctor’s preference, it can be done under general or local anesthesia. The joint is completely replaced with a prosthesis, which may be made out of metal, ceramic, or plastic. If the whole joint is replaced, it is known as a total joint replacement [22]. This is more common with the hip joint and knee joint since they are weight-bearing rather than with the shoulder joint. Very few people are eligible for partial joint replacement. It depends on the patient’s age and the amount of daily activity that the joint is subject to [23,24]. Non-steroidal anti-inflammatory drugs are given to reduce the swelling and pain in the joint to ease the patient. The person must have a healthy diet, physical therapy, blood work, electrocardiogram, and a chest X-ray in the weeks leading up to the surgery [25,26].

RSA is the process where the arthroplasty is done in the shoulder joint, but the parts of the joint are placed in a reverse order to increase the mobility of the shoulder joint [27]. It is a type of shoulder arthroplasty that uses a convex glenohumeral joint. The center of rotation is moved inferiorly and medialized, allowing the deltoid muscle to act on a longer fulcrum and have more mechanical advantage [28,29].

In situations like comminuted four-part fractures of the proximal humerus, cases where previous shoulder arthroplasty has failed and rotator cuff tear arthropathy, RSA is recommended. The benefit of opting for an RSA lies in repositioning the rotational center to a lower and more medial position. Consequently, this alteration permits the deltoid muscle to exert its force over an extended lever, offering a greater mechanical advantage. This adjustment aids in compensating for the weakened rotator cuff muscles, enabling improved shoulder abduction. However, this procedure does not significantly enhance the capacity for the shoulder’s internal or external rotation [30]. The differences between a conventional arthroplasty and RSA are enlisted in Table 1.

**Table 1:** Differences between a conventional arthroplasty and a reverse shoulder arthroplasty The table was created by the authors of this study.

Parameters	Arthroplasty	Reverse shoulder arthroplasty
Anatomical relation	Normal	Reversed
Rotator cuff injury	Repairable	Irreparable
Recurrence	More chances	Less chances
Range of motion	Less than reverse shoulder arthroplasty	More than conventional arthroplasty

Latissimus dorsi transfer in RSA

RSA can be combined with latissimus dorsi transfer to assist with external rotation. Many studies are being conducted to decide the appropriate place for the tendon insertion. The common places are greater tuberosity, the place where teres minor inserts, the upper part where the pectoralis major inserts, etc. Insertion of tendons in all these areas resulted in significantly more torque. The maximum torque was produced by insertion in the greater tuberosity. The torque and external rotation decreased as it was attached more proximal to the humerus. But in most of these studies, the use of deltoid muscle as an active external rotator had not been assessed. These biomechanical advantages are not possible with a routine shoulder arthroplasty since they do not have as much constraint as the RSA. The humeral lateralization was varied in different studies while checking for the benefit of the latissimus dorsi graft in the complete absence of the external rotators. Most of the time, the degrees of torque required are calculated before inserting the latissimus dorsi tendon, and the attachment is done accordingly. The patient is made to lie down by the side, and the arm of the patient is placed in a flexed position. Then, two incisions are made. One of them is at the front, while the other is at the back of the shoulder. The back incision is used to access the latissimus dorsi muscle tendon. The tendon is detached from the bone. A suture is then tied on the free end of the tendon. A flap is created on the deltoid muscle in the front. The tendon is then tied to the rotator cuff muscles, which are not torn or damaged, and the sutures are tightened against the bones of the shoulder joint. The flaps are then closed in the back and at the front end [31,32].

Rheumatoid arthritis (RA) patients frequently experience shoulder cuff tears, with a moderate to severe rate of occurrence. When these tears are present, joint replacement operations have a terrible prognosis. This is caused by several things, including insufficient pain management, instability, uneven wear, superior humeral migration, early failure, glenoid loosening, and higher shear stresses at rates as high as 50%. As a result, the rotator cuff’s health greatly impacts the outcome. Patients with undamaged cuffs experience a greater degree of pain alleviation and improved function. The success of cuff repair during arthroplasty surgery has been reported in several studies. Still, these findings should be interpreted with caution since they may not apply to rheumatoid patients, who frequently undergo unavoidable and progressive upward migration of the head of the humeral bone as a result of cuff failure. Additionally, the subscapularis, infraspinatus, and teres minor muscles are frequently involved in rotator cuff injuries in RA patients. This more extensive cuff involvement could increase the likelihood of instability and produce fewer desirable results [33]. Most people with RA experience moderate to severe erosive alterations to the glenoid (Larsen grades 3-5) and humeral head. Erosion of the glenoid makes implanting a component more difficult and increases the risk of unstable fixation, early implant failure, and less acceptable fixation. In order to avoid problems in restoring the eroded socket, earlier practitioners recommended hemiarthroplasty as the preferred course of treatment for patients with significant glenoid abnormalities. However, after the treatment, the functional results were subpar and got worse with time, primarily because of increased glenoid wear. Despite knowing that hemiarthroplasty in rheumatoid patients with an intact coracoacromial arch may eventually fail, some surgeons still advocate it. It should be mentioned that hemiarthroplasty has been used in conjunction with large allografts like the tendoachilles to stabilize shoulders with rotator cuff and glenoid abnormalities having poor outcomes [34]. The problems in the RA patients might be fixed to some extent with RSA. The biomechanical characteristics of this design boost stability (especially against superior escape) and greatly strengthen the deltoid’s lever arm, which may lead to an increase in abduction strength and range even in the presence of a weak rotator cuff. Despite these biomechanical advancements, professional authors have previously advised against employing RSA in RA patients due to worries about a higher risk of perioperative complications, subpar functional results, and a high revision rate. By merging data from previous series and reporting pooled data on outcomes, complications, and technical factors, this study aims to revisit the evidence supporting these concerns [35].

Indications and contraindications

Anatomic total shoulder arthroplasty (TSA) and reverse total shoulder arthroplasty (RTSA) have become increasingly prevalent procedures for managing arthritic conditions. While TSA is widely embraced for glenohumeral arthritis when the rotator cuff is intact, apprehensions concerning RTSA remain due to varying rates of complications and outcomes [35].

Surgery might be a viable choice if one experiences profound pain that interferes with the daily activities. Additionally, surgical intervention might be recommended if there is shoulder weakness with difficulty in achieving full range of motion. Furthermore, surgery could be suggested if the symptoms fail to improve with alternative treatments like medications, injections, and physical therapy. The primary objective of a latissimus dorsi transfer is to alleviate pain and enhance functionality in cases of irreparable tears in the posterosuperior rotator cuff. This procedure aims to restore the transversal force couple, thereby improving external rotation and preventing the superior migration of the humeral head. Surgical intervention is recommended for individuals experiencing intense shoulder pain and limited external rotation due to a posterosuperior rotator cuff tear that cannot be repaired. Typically, a latissimus transfer is considered when there is noticeable upward movement of the humeral head, significant fatty infiltration of the rotator cuff, and a notable reduction in range of motion and strength. The range of movements include external rotation and abduction. The benefits of using an arthroscopically assisted approach include avoiding detachment of the deltoid muscle from its point of origin, smaller incisions, and the ability to address any additional intraarticular issues [8,32,33,36].

Contraindications arise in cases of extensive posterosuperior rotator cuff tears where there is relatively minimal pain and satisfactory shoulder functionality; a latissimus transfer may not be necessary. Moreover, when subscapularis tears, osteoarthritis, deltoid dysfunction, or shoulder stiffness are present, tendon transfer yields less favorable clinical outcomes [8,36,37].

Lower trapezius transfer can help in an increased range of motion and can also lead to seroma formation. Pectoralis major transfer is preferred in patients with undamaged supraspinatus for a greater pain relief [8]. The benefits and drawbacks of different types of tendon transfers in RSA along with their indications are given in Table 2.

**Table 2:** Benefits and drawbacks of different types of tendon transfers in reverse shoulder arthroplasty The table was created by the authors of this study.

Type of tendon transfer	Indications	Advantages	Disadvantages
Latissimus dorsi transfer	Anterior rotator cuff tears, posterior rotator cuff tears	More external rotation and depression of the head of the humerus due to coupling force on the posterior aspect	Effective in patients with intact subscapularis and less effective in patients with pseudoparalysis of the shoulder; reduction of glenohumeral interval results in an increased risk of glenohumeral arthritis and can lead to axillary hematomas and frozen shoulder
Lower trapezius transfer	Posterior rotator cuff tears	Helps in the same line movement as the infraspinatus, more degrees of range of motion	Can lead to seroma, if subcutaneous tunneling done
Pectoralis major transfer	Anterior rotator cuff tears	Better improvement in the function in patients with undamaged supraspinatus, greater degree of pain relief	No role in improving subscapularis function, not useful in anterior dislocation of the head of the humerus

## Conclusions

Surgical intervention involving joint replacement involves the extraction of the damaged parts of the joint, which are then substituted with resilient artificial components. Although conventional shoulder arthroplasty effectively addresses joint deterioration in numerous patients, its success largely depends on a robust rotator cuff to alleviate pain and enhance the range of motion. However, patients with extensive rotator cuff tears do not experience the same positive outcomes with conventional arthroplasty. Instead, an alternative procedure called RSA is employed. This surgical approach reconfigures the functioning of one's shoulder muscles and takes advantage of the strength of other muscles if one's rotator cuff is compromised. RSA is a better approach than conventional arthroplasty in cases of rotator cuff tears, and it is best implemented if done with a latissimus dorsi transfer. This ensures less pain, greater shoulder strength, and a more mobile shoulder joint, which improves function and reduces the chances of requiring another procedure. RSA has its drawbacks, like failure to do a proper tendon transfer, the tearing of the sutured tendon, weakness in the shoulder joint, and lesser active movement. Furthermore, it cannot be performed in people with severe osteoarthritis or progressive diseases of chondrocytes. However, in indicated patients who do not have an irreparable rotator cuff tear, it can provide a wide range of benefits that would not have been available with conventional arthroplasty surgery.

## References

[1] Yang S, Kim TU, Kim DH, Chang MC (2021). Understanding the physical examination of the shoulder: a narrative review. Ann Palliat Med.

[2] Zeng Z, Liu M, Liu Y (2023). Anatomy features of the shoulder joint in asymptomatic chinese Han adults. BMC Musculoskelet Disord.

[3] Jones L (1956). The shoulder joint—observations on comparative anatomy, physiology, and treatment. Calif Med.

[4] Muench LN, Imhoff AB (2021). The unstable shoulder: what soft tissue, bony anatomy and biomechanics can teach us. Knee Surg Sports Traumatol Arthrosc.

[5] Villaseñor-Ovies P, Vargas A, Chiapas-Gasca K (2012). Clinical anatomy of the elbow and shoulder. Reumatol Clin.

[6] Lin YL, Karduna A (2017). Errors in shoulder joint position sense mainly come from the glenohumeral joint. J Appl Biomech.

[7] Moor BK, Bouaicha S, Rothenfluh DA, Sukthankar A, Gerber C (2013). Is there an association between the individual anatomy of the scapula and the development of rotator cuff tears or osteoarthritis of the glenohumeral joint?: a radiological study of the critical shoulder angle. Bone Joint J.

[8] Clark NJ, Elhassan BT (2018). The role of tendon transfers for irreparable rotator cuff tears. Curr Rev Musculoskelet Med.

[9] Fadul M, von Rotz A, Alsaaod M, Sato R, Steiner A (2020). Arthroscopic approaches to and anatomy of the shoulder joint of cattle: a cadaver study. BMC Vet Res.

[10] Lahti A, Andernord D, Karlsson J, Samuelsson K (2016). [Shoulder dislocation]. Lakartidningen.

[11] Boileau P, Gauci MO, Wagner ER, Clowez G, Chaoui J, Chelli M, Walch G (2019). The reverse shoulder arthroplasty angle: a new measurement of glenoid inclination for reverse shoulder arthroplasty. J Shoulder Elbow Surg.

[12] Tooth C, Gofflot A, Schwartz C (2020). Risk factors of overuse shoulder injuries in overhead athletes: a systematic review. Sports health.

[13] Monica J, Vredenburgh Z, Korsh J, Gatt C (2016). Acute shoulder injuries in adults. Am Fam Physician.

[14] Cools AM, Johansson FR, Borms D, Maenhout A (2015). Prevention of shoulder injuries in overhead athletes: a science-based approach. Braz J Phys Ther.

[15] Kennedy JS, Garrigues GE, Pozzi F (2020). The American Society of Shoulder and Elbow Therapists' consensus statement on rehabilitation for anatomic total shoulder arthroplasty. J Shoulder Elbow Surg.

[16] Jonsson EÖ, Ekholm C, Salomonsson B, Demir Y, Olerud P (2021). Reverse total shoulder arthroplasty provides better shoulder function than hemiarthroplasty for displaced 3- and 4-part proximal humeral fractures in patients aged 70 years or older: a multicenter randomized controlled trial. J Shoulder Elbow Surg.

[17] Smolen JS, Aletaha D, McInnes IB (2016). Rheumatoid arthritis. Lancet.

[18] Lin YJ, Anzaghe M, Schülke S (2020). Update on the pathomechanism, diagnosis, and treatment options for rheumatoid arthritis. Cells.

[19] Jeno SH, Varacallo M (2023). Anatomy, Back, Latissimus Dorsi. https://www.ncbi.nlm.nih.gov/books/NBK448120/.

[20] Alrabaa RG, Ahmad CS (2020). Latissimus dorsi tendon repair. Arthrosc Tech.

[21] Anastasopoulos PP, Alexiadis G, Spyridonos S, Fandridis E (2017). Latissimus dorsi transfer in posterior irreparable rotator cuff tears. Open Orthop J.

[22] Kurowicki J, Triplet JJ, Rosas S, Berglund DD, Horn B, Levy JC (2020). Comparative outcomes of various combinations of bilateral shoulder arthroplasty. Hand (N Y).

[23] Shu B, Ou X, Hu L (2023). Influential articles on shoulder arthroplasty: bibliometric analysis and visualized study. J Shoulder Elbow Surg.

[24] Mauch F, Huth J (2023). [Revision of anatomic shoulder arthroplasty]. Orthopadie (Heidelb).

[25] Ajibade DA, Mourad W, Medina G, Wiater JM (2022). Simultaneous bilateral shoulder arthroplasty: a case series. J Shoulder Elbow Surg.

[26] Hawi N, Tauber M, Messina MJ, Habermeyer P, Martetschläger F (2016). Anatomic stemless shoulder arthroplasty and related outcomes: a systematic review. BMC Musculoskelet Disord.

[27] Ajibade DA, Yin CX, Hamid HS, Wiater BP, Martusiewicz A, Wiater JM (2022). Stemless reverse total shoulder arthroplasty: a systematic review. J Shoulder Elbow Surg.

[28] Boileau P (2016). Complications and revision of reverse total shoulder arthroplasty. Orthop Traumatol Surg Res.

[29] Walker M, Brooks J, Willis M, Frankle M (2011). How reverse shoulder arthroplasty works. Clin Orthop Relat Res.

[30] Marra G, Duralde X (2011). Reverse total shoulder arthroplasty: editorial comment. Clin Orthop Relat Res.

[31] Ortmaier R, Hitzl W, Matis N, Mattiassich G, Hochreiter J, Resch H (2017). Reverse shoulder arthroplasty combined with latissimus dorsi transfer: a systemic review. Orthop Traumatol Surg Res.

[32] Scholten DJ 2nd, Trasolini NA, Waterman BR (2021). Reverse total shoulder arthroplasty with concurrent latissimus dorsi tendon transfer. Curr Rev Musculoskelet Med.

[33] Cho CH, Kim DH, Song KS (2017). Reverse shoulder arthroplasty in patients with rheumatoid arthritis: a systematic review. Clin Orthop Surg.

[34] Lévigne C, Chelli M, Johnston TR, Trojani MC, Molé D, Walch G, Boileau P (2021). Reverse shoulder arthroplasty in rheumatoid arthritis: survival and outcomes. J Shoulder Elbow Surg.

[35] He Y, Xiao LB, Zhai WT, Xu YL (2020). Reverse shoulder arthroplasty in patients with rheumatoid arthritis: early outcomes, pitfalls, and challenges. Orthop Surg.

[36] Chan K, Langohr GD, Welsh M, Johnson JA, Athwal GS (2021). Latissimus dorsi tendon transfer in reverse shoulder arthroplasty: transfer location affects strength. JSES Int.

[37] Wey A, Dunn JC, Kusnezov N, Waterman BR, Kilcoyne KG (2017). Improved external rotation with concomitant reverse total shoulder arthroplasty and latissimus dorsi tendon transfer: a systematic review. J Orthop Surg (Hong Kong).

